# Proanthocyanidins Protect Against Cadmium-Induced Diabetic Nephropathy Through p38 MAPK and Keap1/Nrf2 Signaling Pathways

**DOI:** 10.3389/fphar.2021.801048

**Published:** 2022-01-03

**Authors:** Pin Gong, Peipei Wang, Sihui Pi, Yuxi Guo, Shuya Pei, Wenjuan Yang, Xiangna Chang, Lan Wang, Fuxin Chen

**Affiliations:** ^1^ College of Food and Biotechnology, Shaanxi University of Science and Technology, Xi’an, China; ^2^ School of Chemistry and Chemical Engineering, Xi’an University of Science and Technology, Xi’an, China

**Keywords:** cadmium, diabetic nephropathy, proanthocyanidins, Keap1/Nrf2 signaling pathway, p38 MAPK signaling pathway

## Abstract

Diabetic nephropathy (DN) is one of the most devastating complications of diabetes mellitus. Although cadmium (Cd) exposure might be involved in the pathogenesis of DN, the underlying mechanism is still unclear. In this study, we explored the protective effects and possible mechanism of proanthocyanidins (OPC) from grape seed using a mouse model of Cd-induced DN. The successful establishment of this model was verified by analyzing the physiological and biochemical indices of mice, including their body weight and tissue ratio; levels of blood glucose, creatinine, microalbumin, total cholesterol, triglycerides, high-density lipoprotein-cholesterol and low-density lipoprotein-cholesterol; and was based on histopathological examination. Oxidative-antioxidative status, elemental analysis, and key signaling pathway analysis were performed to explore the possible protective mechanism of OPC. The protective effects of OPC and its possible mechanism in preventing the progression of DN were investigated using a multidimensional approach, including its ability in regulating oxidative-antioxidative status (lipid peroxidation, protein carbonyl, superoxide dismutase, and glutathione GST, GSH-Px), metal-binding ability (Cd levels in the kidneys and urine and MT content) and mediation of essential elements (Zn, Ca, Cu, and Fe levels in the kidneys), and activation of the p38 MAPK and Keap1/Nrf2 signaling pathways. OPC exhibited a significant renoprotective effect, attributed to the metal-chelating ability, anti-oxidative effect, and mediation of oxidative stress-related signaling pathway. These results highlight the potential of OPC in preventing or treating DN in humans and suggest the dietary intake of grapes, which are rich in polyphenols, for the prevention of type 2 diabetes mellitus and its complications.

## Introduction

Diabetic nephropathy (DN) is the most devastating complication of diabetes mellitus that causes significant morbidity and mortality (Zoja et al., 2020). It is gradually becoming a leading cause of end-stage renal disease (Duran-Salgado and Rubio-Guerra, 2014; Hansrivijit et al., 2021). Epidemiological studies reveal that more than 25% of patients with diabetes suffer from DN (Reidy et al., 2014), which is characterized by a series of renal abnormalities including mesangial expansion, basement membrane thickening, tubulointerstitial fibrosis, and glomerulosclerosis (Reidy et al., 2014; Li et al., 2021; Medina Rangel et al., 2021). These changes result in a clinical presentation characterized by hypertension, proteinuria, and a progressive reduction in kidney function (Reidy et al., 2014).

Although several studies have shed light on the pathogenesis of DN, several aspects of its condition remain unclear; thus, further comprehensive investigations and evidence-based findings are needed to elucidate the mechanism underlying disease progression (Mantovani and Zusi, 2020; Miao et al., 2021a). Cadmium (Cd), a transition metal that is, toxic by nature and an environmental pollutant, is generated from industrial sources and found in air, water, food, and cigarette smoke. The primary target organs for Cd toxicity are the liver and kidneys. Epidemiological and experimental studies suggest that Cd may act as a risk factor in the pathogenesis of diabetes mellitus and renal disease (Edwards and Ackerman, 2016). A dose-response relationship has been reported between urinary Cd and albuminuria in Torres Strait subjects with type 2 diabetes. It has been suggested that Cd-mediated toxicity may intensify the effects of diabetes on the kidneys. Exposure to Cd could lead to hyperglycemia and accelerate the progression of diabetes mellitus (Tangvarasittichai et al., 2015). Cd accumulation in the kidneys results in generalized dysfunction accompanied by polyuria, glucosuria, and low-molecular-weight proteinuria (Madrigal et al., 2019). Previous studies suggest that Cd can accelerate the occurrence of DN in male Kunming mice treated with a high fat and sugar diet (HFSD) (Gong et al., 2017).

The molecular pathogenesis of DN is poorly understood and no new drugs have been approved for the therapy of DN in almost 20 years. Thus, there is an urgent need for the screening and identification of novel and efficacious drug candidates for the management of DN. Natural products has long been used to be an alternative therapy for the prevention and treatment of various renal diseases including DN (Chen et al., 2018a; Wang et al., 2018a; Chen et al., 2018b; Yang and Wu, 2021). Therefore, Natural products were considered as an important source of new drugs over past 4 decades (Izzo et al., 2020; Newman and Cragg, 2020; Miao et al., 2021b; Zhou et al., 2021). Proanthocyanidins (OPC) are condensed tannins that are derivatives of catechin and gallic acid that are composed of propelargonidins, procyanidins, and prodelphinidins. These compounds are widely distributed in grapes, green and black teas, grape seeds, and wine (Yokozawa et al., 2012). Of late, OPC have been receiving increased attention owing to their potential health benefits, such as their antioxidant properties with strong radical-scavenging, blood glucose lowering, anti-apoptotic, and anti-inflammatory properties among other biological activities (Yokozawa et al., 2012; Ruan et al., 2020). As OPC are known to reduce blood glucose levels, balance oxidative stress, and ameliorate inflammatory responses, we wanted to further explore if they could play a role in protecting against Cd-induced DN. To our knowledge, there lack studies that have elucidated the protective effects on Cd-induced DN in mice. Therefore, we hypothesized that if OPC could effectively protect against Cd-induced DN, a diet comprising this natural compound may serve as potential therapeutic agent and prevent Cd-induced renal damage.

One of the challenges in drug discovery, especially in the case of diabetes, is the lack of robust animal models that can mimic key features of human DN. In this study, we established a mouse model of DN using Cd exposure and HFSD treatment. The successful establishment of this model was verified based on physiological and histopathological investigations and determining the biochemical indices of mice. We investigated the protective effect and potential mechanism of OPC against Cd-induced DN. This study may provide useful clues for better understanding the etiology of DN and highlight novel insights regarding the use of OPC in a clinical setting.

## Methods

### Materials and Reagents

OPC (purity > 99.50%) were purchased from Yifang S&T Co. Ltd. (Tianjin, China). Cadmium chloride and β-actin (sc-4778, Santa Cruz, CA) were purchased from Sigma-Aldrich Chemical Company (St. Louis, MO, United States) and stored at 4°C away from sunlight. Anti-MAPK (sc-393609, Santa Cruz, CA), anti-p38 (sc-81621, Santa Cruz, CA), anti-nuclear factor-erythroid-2-related factor 2 (Nrf2, sc-365949, Santa Cruz, CA), anti-Keap1 (sc-515432, Santa Cruz, CA), and horseradish peroxidase (HRP)-conjugated secondary antibodies (anti-rabbit) were obtained from Santa Cruz Biotechnology (CA, United States).

### Animals Handing and Sample Preparation

Five-week-old male Kunming mice (weighing 20 ± 2 g) were supplied by Central Animal House, Xi’an JiaoTong University Health Science Center (Xi’an, China). The animals were housed in a specific pathogen free environment at a temperature of 22 ± 1°C and relative humidity of 50 ± 1%, and subjected to a 12/12 h light/dark cycle. All animals were provided free access to food and water and were allowed to acclimatize for 7 days prior to dosing. All animal studies complied with the regulations and guidelines of the Shaanxi University of Science and Technology Institutional Animal Care and were in accordance with the IACUC and AAALAC guidelines.

For experimental studies, the mice were randomly divided into the following four groups with 10 mice per group: control, DN, OPC, and HFSD. The mice in the control and HFSD groups were fed a standard pellet diet or HFSD and intraperitoneally (i.p.) administered physiological saline (0.9%) during the entire procedure. For the initial 12 weeks, mice in the DN and OPC groups were fed HFSD and received a daily injection of 1 mg/kg CdCl_2_ i.p. After treatment for 12 weeks, mice in the OPC group were administered OPC i.p. at a dose of 5 mg/kg body weight for consecutive 4 weeks. Mice in the DN group received 0.9% saline i.p. A detailed experimental protocol is listed in Table 1 and was used in our study (Gong et al., 2017).

**TABLE 1:** Detail protocols for model construction.

Group	Diet	Treatment	
		12 weeks	4 weeks
Control Group (Con)	standard pellet diet	0.9% saline i.p.	0.9% saline i.p.
DN group (DN)	High sugar and Fat diet	1 mg/kg CdCl_2_ i.p.	0.9% saline i.p.
OPC treatment Group (DN + OPC)	High sugar and Fat diet	1 mg/kg CdCl_2_ i.p.	5 mg/kg bw OPC
High Fat and sugar Group (HFSD)	High sugar and Fat diet	0.9% saline i.p.	0.9% saline i.p.

During the entire experimental period, the body weight and fasting blood glucose (FBG) levels of mice were recorded at regular intervals. Using metabolism cages, 24 h urine samples were collected for analysis. Mice were anesthetized by diethyl ether inhalation and sacrificed using cervical dislocation (Pavel et al., 2020). Blood samples were collected from the eyeballs of mice, centrifuged at 4°C to obtain serum, and frozen at −80°C. Kidneys were dissected and weighed immediately, washed clean, and either stored at −80°C or fixed in 10% formalin and stored until further use.

Renal tissues were accurately weighed, cut into pieces, and homogenized in a pre-cooled Teflon homogenizer with an appropriate amount of ice-cold Tris–HCl buffer (100 mM; pH 7.4) [sample weight (g):buffer volume (ml) = 1:9] to obtain the tissue homogenate. The supernatant was centrifuged at 12,000 rpm for 20 min at 4°C to obtain samples for subsequent assays.

### Assessment of Blood and Urine Parameters

Fasting blood glucose (FBG) levels were determined using a glucometer and mice with FBG levels of more than 11.1 mmol/L were considered diabetic. Creatinine and microalbumin (mALB) levels in the urine were analyzed using the corresponding assay kits. Total cholesterol (TC), triglyceride (TG), high-density lipoprotein-cholesterol (HDL-C), and low-density lipoprotein-cholesterol (LDL-C) levels in the blood were determined based on enzymatic assays using commercial kits (Jiangchen Co., Nanjing, China) and following the manufacturer’s instructions.

### Renal Histopathology

For renal histopathological studies, the kidney sections from each mouse were fixed in 10% formalin immediately after dissection and washed using a gradient concentration of ethanol for dehydration, as previously described (Miao et al., 2020). The slides were transparentized with xylene, dipped in wax, and embedded in paraffin. The blocks were sectioned and stained with hematoxylin-eosin (H&E) to observe the normal cellular structure. Periodic acid-Schiff (PAS) staining was used to determine the glycogen content and Masson’s trichrome staining was used to analyze collagen deposition. The stained sections were covered with a glass slide, dried, and observed using an optical microscope (Leica DM750).

### Assessment of Antioxidative Status

Proteins in the kidney tissue homogenates were determined using a bicinchoninic acid (BCA) assay. The oxidative-antioxidative status in mouse kidneys was determined based on the malondialdehyde (MDA), protein carbonyl (PCO), superoxide dismutase (SOD), glutathione (GSH), glutathione S transferase (GST), and glutathione peroxidase (GSH-Px) levels using commercially procured kits. Renal nitric oxide (NO) levels were determined using a colorimetric method based on the Griess reaction.

### Determination of Essential Elements and Metallothionein in Kidney

To quantify the content of essential elements, renal samples were accurately weighed and dry-ashed using a muffle furnace. The ash was solubilized in 3M HCl and appropriately diluted. The serum and urine samples were also pre-deposited, respectively. Tissue, serum, and urine samples were tested for Zn (213.9 nm), Fe (248.5 nm), Cu (324.8 nm), Ca (422.7 nm), and Cd (228.8 nm) using atomic absorption spectrometry (Hitachi Z-2000). The element levels are expressed as micrograms of the element per gram of wet tissue weight (μg/g w.t.w.) or micrograms of the element per milliliter sample (μg/ml) (Gong et al., 2008). MT content in mouse kidney was determined following the methods published by Gong et al. (2012).

### Western Blotting Analysis

Western blotting analysis was performed following previously published studies (Chen et al., 2019a). RIPA buffer was used for the extraction of total protein. Renal tissues were first weighed, mixed with RIPA buffer supplemented with protease and phosphatase inhibitors (Roche Applied HEART), and homogenized at 4°C. The supernatant was centrifuged at 4°C and 13,000 × *g* for 30 min and transferred to a new enzyme-free tube. Proteins were quantified using a BCA kit.

Western blotting was performed using anti-MAPK (Santa Cruz, CA), anti-p38 (Santa Cruz, CA), anti-Nrf2 (Santa Cruz, CA), anti-Keap1 (Santa Cruz, CA), anti-actin (Santa Cruz, CA), and HRP-conjugated secondary antibodies (anti-rabbit) from Santa Cruz. Proteins were separated using electrophoresis on an SDS-polyacrylamide gel and the bands were electrophoretically transferred onto a polyvinylidene difluoride membrane. The membranes were blocked for 1 h at 37°C and incubated with primary antibodies overnight at 4°C followed by hybridization with HRP-conjugated secondary antibodies for 1 h. Proteins were detected using the enhanced chemiluminescence (ECL) system and ECL Hyperfilm (Amersham Pharmacia Biotech, UK Ltd., Little Chalfont, Buckinghamshire, UK). The intensities of relative bands were quantified using densitometry.

### Statistical Analysis

All values are expressed as mean ± standard deviation. All statistical tests were performed using SPSS version 20.0. Significant differences among the different groups were evaluated using one-way analysis of variance (ANOVA) followed by Dunnett’s post hoc test. *p* < 0.05 was regarded significant differences.

## Results

### Effects of OPC on Metabolic Dysfunction in Cd-Induced DN Mice

**FIGURE 1:**
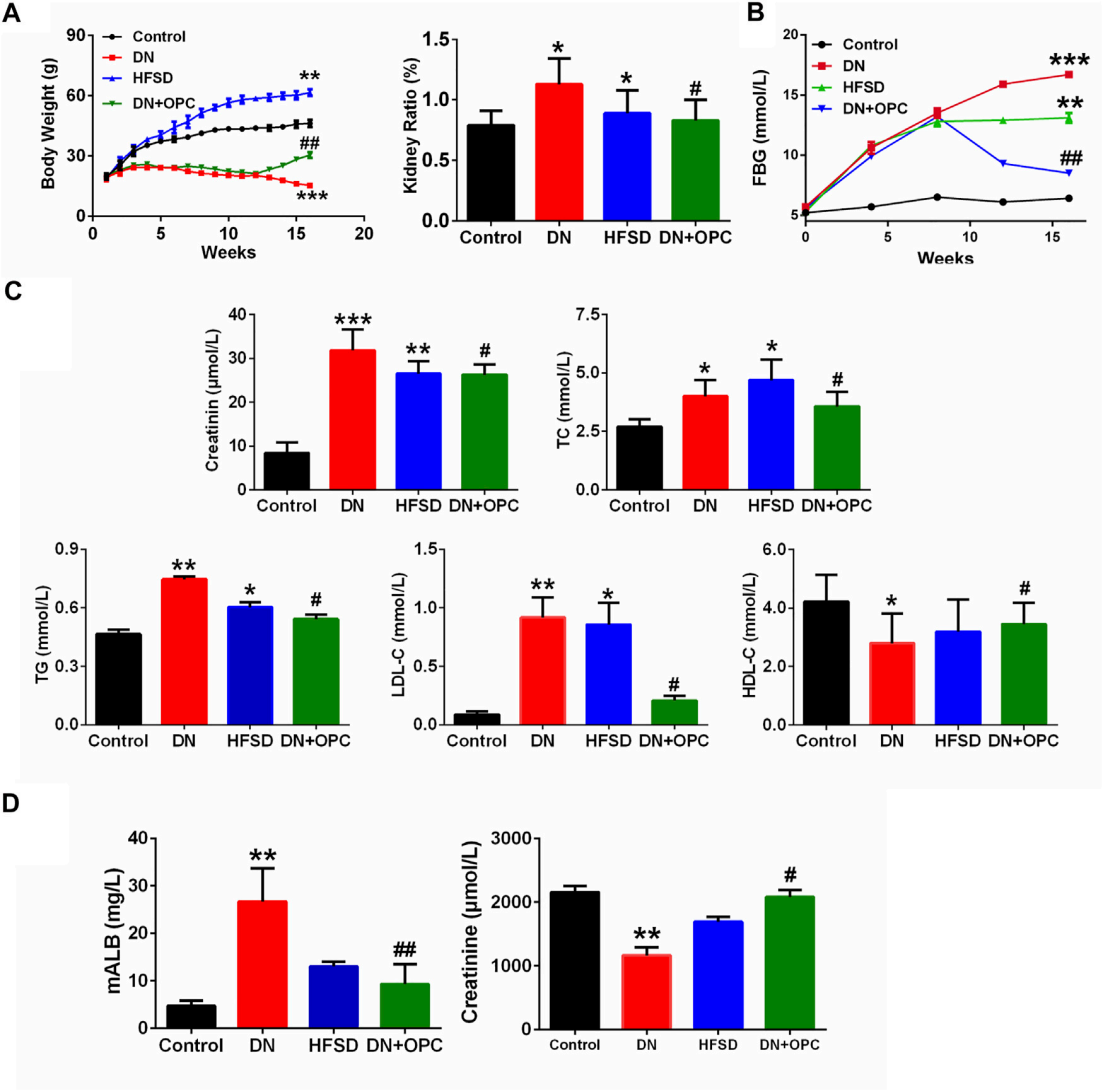
Physiological and biochemical indexes **(A)** The body weight and kidney ratio in the different groups at week 16. kidney ratio equal to kidney weight/body weight. **(B)** FBG in the different groups. **(C)** Serum parameters in the different groups. Including serum creatine, TC contents, TG contents, LDL-C contents, and HDL-C contents in the different groups. **(D)** mALB contents and Urine creatinine in the different groups. *p < 0.05, **p < 0.01, ***p < 0.001 compared with control group; ^#^ *p* < 0.05, ^##^ *p* < 0.01, compared with DN group.

The body weight gain of DN mice was significantly decreased compared with the control mice. We found that the administration of OPC for 12–16 weeks could recover this decline (*p* < 0.05). When mice were treated only with HFSD, significant increases in body weight were observed (Figure 1A). Compared with the control group, the tissue ratio was significantly increased by 43.1% in the DN group and treatment with OPC could decrease this ratio. Moreover, the tissue ratio in the HFSD group also showed a significant increase of 12.7% compared with the control mice (Figure 1A).

### Effects of OPC on Glucose and Lipid Metabolism in Cd-Induced DN Mice

FBG levels were also measured during the entire study. As shown in Figure 1B, in the initial 12 weeks, when treated with HFSD and/or Cd, the FBG levels of mice in the DN, OPC, and HFSD groups were all above 11.1 mmol/L, indicating that the animals were diabetic. The administration with OPC caused a significant decrease in FBG (*p* < 0.01), suggesting its possible role in lowering FBG. To determine the protective effects of OPC in mice with Cd-induced DN, the serum biochemical parameters, such as plasma TG, TC, HDL-C, and LDL-C, related to *in vivo* glucose and lipid metabolism were evaluated, and the results are presented in Figure 1C. The levels of TG, TC, and LDL-C of mice in the DN group were markedly increased, whereas HDL-C levels were significantly decreased compared with the control group. On the other hand, TG, TC, and LDL-C levels were found to be significantly decreased and HDL-C level was significantly increased in mice treated with OPC compared with those in the DN group. Additionally, the changes in TC, TG, HDL-C, and LDL-C levels of mice in the HFSD group were moderate, probably because Cd exposure accelerated the progression of DN.

### Effects of OPC on the Renal Function in Cd-Induced DN Mice

Next, we determined renal function and evaluated the protective effects of OPC against Cd- and HFSD-induced DN. The combined effect of Cd and HFSD led to remarkable renal dysfunction as evidenced by serum/urine creatinine and urine mALB levels (Figures 1C,D). Plasma and urine creatinine levels were determined to assess the degree of renal injury. As shown in Figure 1C, plasma creatinine levels were remarkably elevated in mice in the DN group compared with the control mice. Creatinine levels of mice in the OPC group were 17.5% lower than those in the DN group. Of note, the change in urine creatinine levels showed an opposite trend; creatinine levels of mice in the DN group were remarkably decreased to 54% vs. those in the control group. However, OPC treatment effectively ameliorated this change and restored to near normal levels.

We also found that compared with the control group, urine mALB levels of mice in the DN group were significantly increased. OPC treatment greatly reduced mALB levels to 65.1% compared with those observed in the DN group (Figure 1D). These results indicated the protective effects of OPC against the development of DN.

### Effects of OPC on the Renal Histology in Cd-Induced DN Mice

**FIGURE 2:**
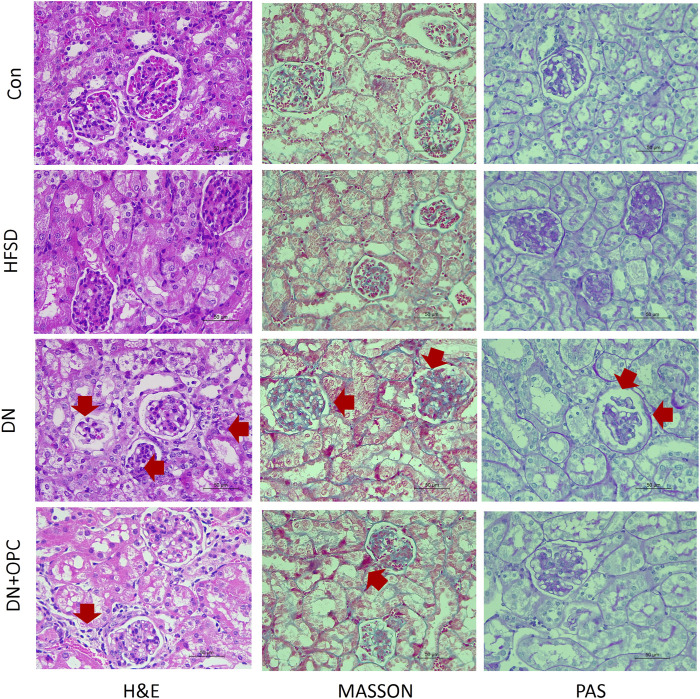
Pathologic analysis of the kidneys. H&E staining in the different groups, including control, HFSD, DN, and DN + OPC groups, (*) represent glomerulus atrophy, (→) represent renal tubular necrosis. PAS staining in the different groups, including control, HFSD, DN, and DN + OPC groups, (→) represent mesangial expansion. MASSON staining in the different groups, including control, HFSD, DN, and DN + OPC groups. (→) represent inflammatory infiltrates, areas of tubulointerstitial fibrosis. Magnification times × 400 and scale bar in 50 μm.

Kidney histology was studied using H&E staining, which revealed significant changes as glomerular thickening in the tissue samples of mice from the DN group (Figure 2). Interstitial and glomerular changes were noticeably ameliorated after OPC treatment (H&E staining, Figure 2). A diffuse expansion of the mesangial matrix and prominent thickening of the glomerular basement membrane to a mild extent was observed in samples from the DN group after PAS staining, which was greatly reduced after OPC treatment.

Moreover, renal histology was studied using Masson’s staining (Figure 2), which demonstrated visible changes in the tissues from the DN groups, as evidenced by mild tubulointerstitial fibrosis, accumulation of extracellular matrix (ECM), tubular dilatation, and atrophy. OPC treatment was found to partly reduce the tubulointerstitial fibrosis index in the renal cortex and medulla. Collectively, these results indicated the protective role of OPC in Cd-induced DN by the amelioration of morphological changes.

### Effects of OPC on Renal Oxidative Stress Parameters and NO Levels in Cd-Induced DN Mice

**FIGURE 3:**
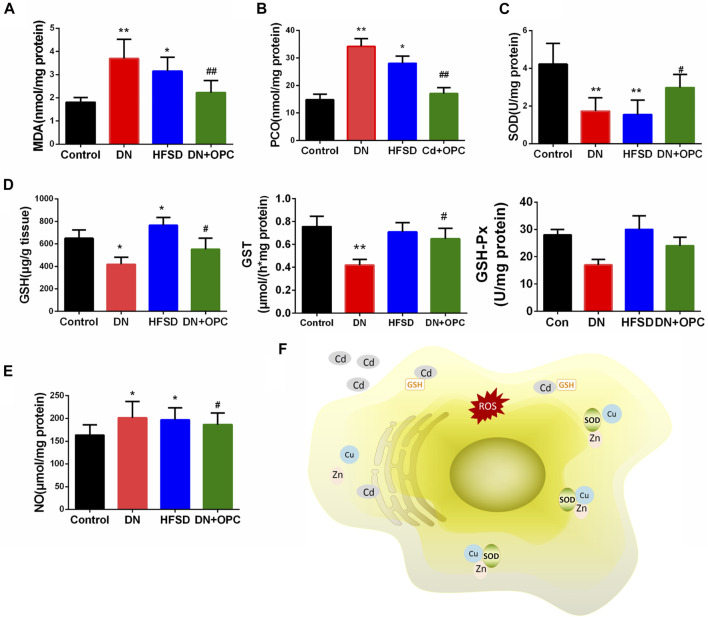
Anti-oxidative parameters of the kidneys. **(A)** MDA levels. **(B)** PCO levels. **(C)** SOD activities. **(D)** GSH, GST, and GSH-Px content. **(E)** NO levels. **(F)** Possible mechanism of cadimium on oxidative stress. *p < 0.05, **p < 0.01, ***p < 0.001 compared with control group; and ^#^ *p* < 0.05, ^##^ *p* < 0.01, compared with DN group.

As the pathology of DN is related to the overproduction of ROS and an imbalance in oxidative stress, we determined the oxidative stress parameters. As shown in Figures 3A,B, the MDA and PCO levels showed a 2.1-fold and 2.3-fold increase in the DN group, respectively, after exposure to Cd and HFSD. OPC treatment could attenuate oxidative stress by significantly decreasing MDA and PCO levels. As expected, the synergistic effect of Cd and HFSD resulted in a 59.1% decrease in SOD activities in mice with DN (*p* < 0.01), whereas OPC treatment could effectively reverse this decrease (Figure 3C).

We also found that compared to that in the control animals, the combined effect of Cd and HFSD could reduce GSH, GST and GSH-Px levels by 35.5, 44.4, and 39.2% in mice in the DN group, respectively, whereas OPC treatment significantly increased GSH, GST and GSH-Px levels (Figure 3D). Moreover, NO levels in the tissues of mice from the DN group were significantly increased co-treatment with Cd and HFSD (Figure 3E). However, NO levels were found to be reduced in tissues in the OPC group. Treatment with only HFSD led to significant changes in the oxidative and antioxidant parameters except for the NO levels.

### Effects of OPC on p38 MAPK and Keap1/Nrf2 Pathway-Mediated Oxidative Stress

**FIGURE 4:**
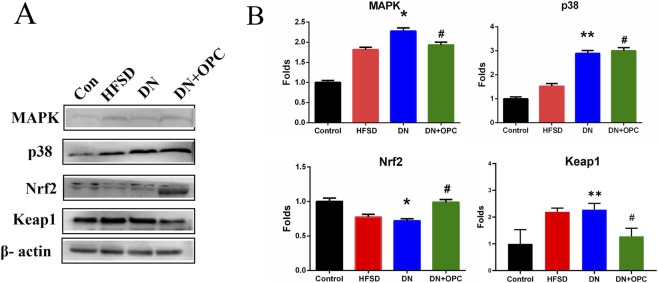
Western blot analysis. **(A)** Protein Bands. **(B)** Quantitative analysis. Data were repeated three times. *p < 0.05, **p < 0.01, compared with control group and ^#^ *p* < 0.05 compared with DN group.

Western blotting was used to further confirm the role of oxidative stress in the progression of DN and to determine the protective mechanism of OPC in oxidative stress-related protein expression. Accordingly, we examined the protein expression of the p38 MAPK and Keap1/Nrf2 pathways. Our findings indicated that p38 and MAPK expression significantly increased in DN kidney samples; however, this increase was prevented after treatment with OPC (Figure 4).

There was a significant decrease in Nrf2 expression and an increased expression of Keap1 in the samples obtained from the DN group. However, treatment with OPC significantly increased Nrf2 and decreased Keap1 expression in diabetic conditions (Figure 4). These results suggested that OPC may exert protective effects in DN, which might be associated with p38 MAPK and Keap1/Nrf2 signaling pathways.

### Effects of OPC on Elemental Levels in Cd-Induced DN Mice Kidneys

**FIGURE 5:**
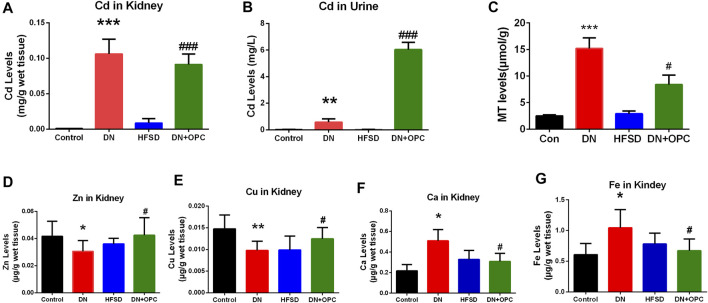
Elemental analysis and MT content determination. **(A)** Cadmium content in kidney. **(B)** Cadmium content in urine. **(C)** MT content. **(D)** Zinc content in kidney. **(E)** Copper content in kidney. **(F)** Calcium content in kidney. **(G)** Iron content in kidney. ***p < 0.001, **p < 0.01, *p < 0.05 compared with control group and ^#^ *p* < 0.05, ^##^ *p* < 0.01, ^###^ *p* < 0.001, compared with DN group.

An imbalance in elemental levels is common in patients with DN. Thus, we investigated the changes in levels of the essential elements in DN and the effect of OPC on elemental levels. It can be seen in Figure 5 that Cd was accumulated in the kidneys of mice in the DN and OPC-treated groups. A significant elevation of Cd levels was observed in samples obtained from the DN group (111.1-fold higher than that in the Control group; *p* < 0.001, compared to the Control group). This accumulation was reduced after OPC treatment and Cd levels in the kidneys were found to be reduced to 85.9% compared with those in the DN group. Cd was excreted in the urine in a small amount. OPC treatment led to a significant increase in Cd excretion (10.4 folds higher than that in the DN group; *p* < 0.01, compared with the DN group). There was no significant increase in Cd levels in the kidneys or urine of samples obtained from the HFSD group.

As seen in Figure 5, levels of the essential elements were significantly different among groups. Zn levels in the kidney tissues of mice from the DN group were significantly decreased to 73.3%, and OPC could restore these levels.

Cu levels showed a 1.34-fold increase in the tissue samples obtained from mice in the DN group compared with those from the control group. OPC could ameliorate this increase and return to normalcy. Ca and Fe levels in the tissue samples in the DN group showed a 2.35-fold and 1.72-fold increase, respectively, whereas OPC could effectively decrease this tendency and reduce Ca and Fe levels to 60.5 and 64.1%, respectively. Changes in the elemental levels in the HFSD group were not significant.

To further elucidate the mechanism of OPC chelating with cadmium, the content of MT in kidney were assessed. As shown in Figure 5, an increase of MT content in DN group was observed (*p* < 0.001), whereas the treatment of OPC could lower the content of MT, suggesting the binding ability of OPC with cadmium.

## Discussion

The progression of DN, one of the biggest problems in nephrology field, pointing to that patients progress end-stage renal disease and require dialysis (Aydin et al., 2019; Bacharaki et al., 2021) or kidney transplantation (Webster et al., 2017; Jain et al., 2019; Van Sandwijk et al., 2019). Recent epidemiological studies reveal a link between Cd exposure and the incidence of diabetes and its complications (Chen et al., 2006; Afridi et al., 2008; Akinloye et al., 2010; Satarug et al., 2010; Wallin et al., 2014). Several hypotheses have been proposed to explain the interplay between Cd exposure and incidence of diabetes and DN (Edwards and Prozialeck, 2009; Park et al., 2019). Gong *et al.* suggest that Cd might be a risk factor in DN pathology (Gong et al., 2017); however, the underlying mechanism of the role of Cd in the development and progression of DN is yet to be completely elucidated. In this study, we investigated the possible etiology of Cd-induced DN and studied OPC as novel and efficacious drug candidates in DN therapy.

A decrease in body weight in patients with diabetes may be due to the compensatory gluconeogenesis in cells to account for glucose deficit. Thus, weight reduction may be considered a marker of diabetes mellitus and its complications. The body weight reduction in DN mice is probably due to abnormal carbohydrate and lipid metabolism and increased protein catabolism (Ghosh et al., 1994). With the onset of DN, the kidney size and weight increased by an average of 15% and was accompanied by a progressive reduction in renal function. The increase in kidney weight was likely due to the development of renal hypertrophy. OPC are known to effectively alleviate kidney enlargement (Mansouri et al., 2011). Elevated FBG levels in mice with DN suggested the diabetogenic effect of Cd also revealed that the administration of OPC could effectively reduce the spike in blood glucose.

We also observed an increase of mALB levels in the DN group, which probably resulted from the damage to the renal glomeruli filtration barrier; however, the precise mechanism of this phenomenon is still unknown. A plausible explanation is that the severity of pro-oxidative stress in diabetes results in disturbed metabolism in the basement membrane (Ha and Kim, 1995). mALB is the predominant renal risk marker in patients with DN. The high mALB levels exacerbate renal damage. A decrease in mALB results in a proportional increase in renal function. Moreover, the creatinine in urine decreased as the concentration of creatinine in the blood was acutely increased 6–8 folds by creatinine infusion. This decrease was thought to be due to the saturation of the tubular secretory mechanism (Perrone et al., 1992). The renal glomeruli from DN mice showed a characteristic morphology that correlated with mesangial cell proliferation and excessive accumulation of ECM (Ahn et al., 2004). We found that OPC exerted a protective effect, prevented the increase in mALB and creatinine levels, and retained renal morphology. Our findings were in agreement with the results of the study by Stefanovic *et al.* wherein grape seeds, which are rich in antioxidative bioflavonoids, were found to bring about a significant morphologic improvement and amelioration in kidney function (Stefanovic et al., 2000).

The unusually high levels of serum lipids are probably due to the increased mobilization of free fatty acids from the peripheral fat deposits, as insulin suppresses the production of hormone-sensitive lipase (Sugano et al., 2006; Vaziri, 2006). Therefore, elevated TG, TC, and LDL-C levels that were found in the DN group may also constitute risk markers during the development of DN. We found that OPC could abate the susceptibility of lipids to oxidation and stabilize the membrane lipids, thereby relieving oxidative stress by exerting hypocholesterolemic, hypotriglyceridemic, and hypophospholipidemic effects.

**FIGURE 6:**
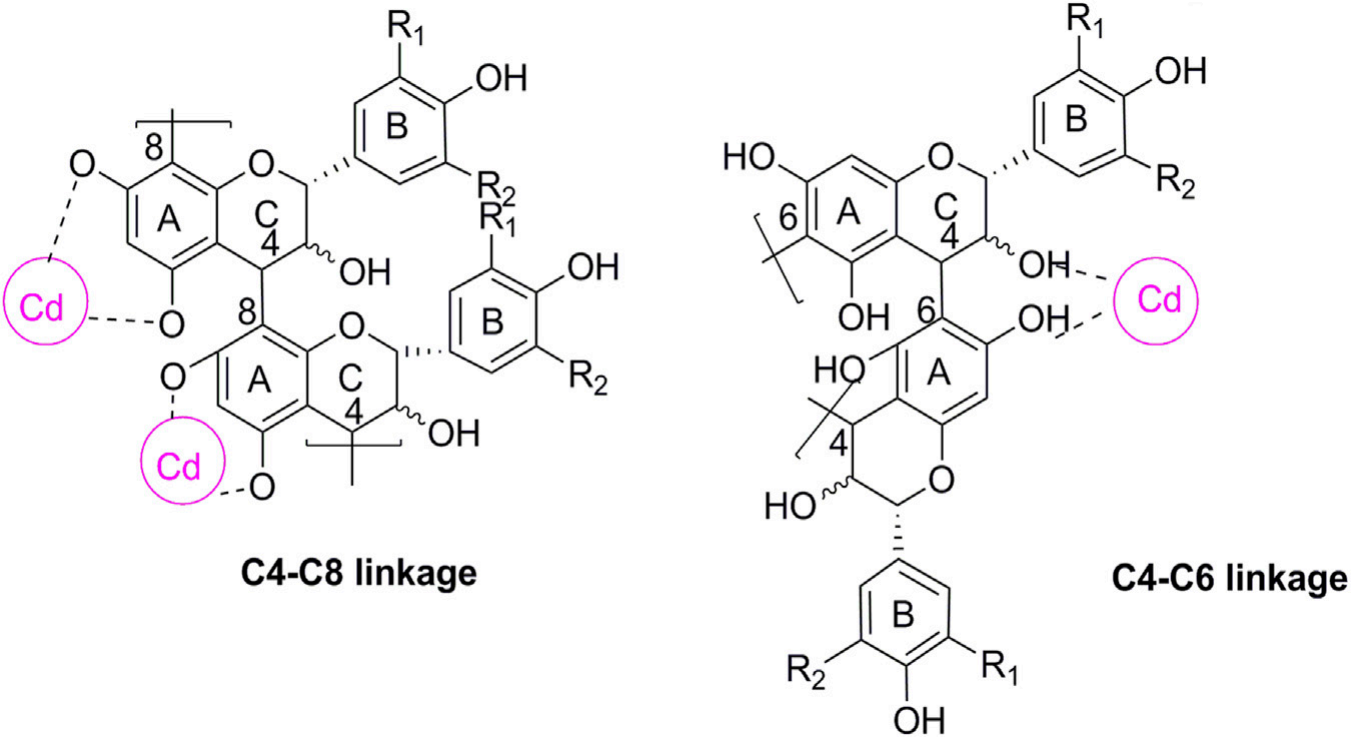
Chemical structure of proanthocyanidin-cadmium polymers. R1, R2 = H, propelargonidins; R1 = OH, R2 = H, procyanidins; R1, R2 = OH, prodelphinidins. The cycle in purple represent the possible binding site for Cd.

The significant increase in Cd levels in the tissue samples from the DN group suggested Cd accumulation, especially in the kidneys. The toxicity of Cd may be partly attributed to its long half-life and low excretion rate. The urine Cd levels were found to be remarkably elevated after OPC treatment, possibly suggesting the metal-chelating ability of OPC in reducing Cd levels *in vivo.* Grape seeds are a rich source of plant flavonoids and OPC oligomers. Proanthocyanidins from grape seed are polyphenolic compounds that are mainly concentrated in tree barks and outer skin of the seeds. OPC is of a diphenylpropane structure of C6 -C3 -C6. Most often found as a glycoside derivative, this compound class is composed of three monomer units of catechin, epicatechin, and epigallocatechin (Harborne and Mabry, 2013). OPC bind metals through complexation *via* their o-diphenol groups. Fine et al. found that OPC can chelate free iron molecules, and inhibit iron-induced lipid peroxidation (Fine, 2000). As seen in Figure 6, OPC can bind with Cd ions to form a covalent structure that can be excreted *via* urine.

Enteric neuropathy and microvascular disease affect the *in vivo* absorption of essential minerals in diabetic individuals (Eliasson et al., 1995). Cd competes with essential elements such as Ca, Zn, Fe, and Cu, thereby resulting in an imbalance. Low Zn levels may interfere with the ability of the pancreatic islet cells to produce insulin, especially in individuals with type 2 diabetes. Moreover, a decrease in Zn is linked to the overproduction of free radicals and increased lipid oxidation in patients with diabetes (DiSilvestro, 2000). Moreover, Zn deficiency may exacerbate the toxicity of other metals such as Fe and Cu. During hyperglycemia and inflammation, excess Fe results in the overproduction of ROS and may affect glucose control, leading to the development and progression of DN (Al-Hafidh Khattab and Al-Youzbaki, 2018). Intracellular calcium levels are increased in patients with diabetes. Ca^2+^ release in the cytosol accelerates Cd-induced mitochondrial injury, thus mediating cytochrome-c release and caspase-9 activation. Increased Cu levels have been correlated with the oxidation of LDL-C and alterations in arterial wall structure, eventually leading to infection, stress, and diabetes mellitus (Beshgetoor and Hambidge, 1998; Tan et al., 1999). Since MT is belived to play a privotal role in the detoxication of heavy metals, such as cadmium, and scavenging of free radicals, the metal ability of OPC were assessed by measuring the content of MT. It is obvious that OPC treatment could lower the content of MT, suggesting the protective of OPC against Cd-induced nephrotoxicity probably due to its ability to chelating metal ions (Gong et al., 2012).

When chronically exposuring to Cd, approximately 50% of the absorbed Cd is accumulated in the kidneys and causes damage to renal microtubules (Satarug et al., 2017). However, the mechanism for cadmium nephrotoxicity remains uncertain. Using a variety of cell culture systems or animal models, ROS is thought to be associated with cadmium toxicity. Cadmium depletes glutathione and protein-bound sulfhydryl groups, leading to the production of reactive oxygen species, such as superoxide anions, hydrogen peroxide, and hydroxyl radicals. The depletion in renal GSH has been observed in response to oxidative stress by cadmium treatment, resulting in enhanced lipid peroxidation, and excessive lipid peroxidation caused increased GSH consumption. As GSH-dependent antioxidant enzymes, the decrease abilities of GST and GSH-Px probably due to reduced content of its substrate GSH. The detoxification effect of GST play a critical role for endogenous compounds such as peroxidised lipids, as well as the metabolism of xenobiotics. GSH-Px could eliminate hydrogen peroxide and lipid peroxides and thus interrupt the propagation of the lipid peroxidation reaction (Valavanidis et al., 2006). Moreover, the exposure to cadmium lead to the loss of Cu and Zn in the kidneys. Cu and Zn are antioxidant trace elements because they act as the cofactors of cytoplasmic superoxide dismutase (Pokusa and Kráľová Trančíková, 2017). The decreased SOD activity might be related to the loss of copper and zinc which are essential for the enzyme activity, resulting in the insufficient abilities to scavenge the superoxide anion produced during the normal metabolic process. Hamasaki *et al.* found that an imbalance between Zn and Cu is associated with renal dysfunction in humans, which is mediated by oxidative stress (Hamasaki et al., 2016). The increased production of different radical species accelerates the degradation of carbohydrates, lipids, and DNA, and further result in hyperglycemia and glucose auto-oxidation (Gong et al., 2017). As a result, oxidative stress induced by Cd may be an intervening factor in the toxic mechanism of Cd on renal tubules (Figure 3F).

Hyperglycemia-induced oxidative stress might also act as a common but key event in the development and progression of diabetes and its complications (Ceriello, 2000). Decreased SOD activity has been reported in diabetes (Mohora et al., 2006). Depletion of reduced GSH could significantly affect the overall redox potential of the cell. NO reacts with superoxide radicals and is converted to the harmful peroxynitrite, leading to increased levels of steady-state free radicals in the kidneys of diabetic individuals (Huie and Padmaja, 1993). Stadler *et al.* have demonstrated that NO production is increased in the kidneys of streptozocin-induced diabetic rats, which provides credible evidence for the involvement of ROS, NO, and peroxynitrite-derived species in the development and progression of early diabetic tissue damage (Stadler et al., 2003). OPC as free radical scavengers can increase free GSH levels to further detoxify the products of lipid peroxidation (Bagchi et al., 2000), and then restoring the renal antioxidant defense system. OPC also promote insulin release by stimulating the surviving pancreatic cells and regulating insulin release, thereby facilitating serum glucose levels to normalcy. In the present study, combined treatment of cadmium and HFSD resulted in significant increase in TBARS and PCO as well as NO, significant decrease in antioxidant content in renal tissue, however, treatment with OPC could ameliorate these changes, indicating the protection offered by OPC against renal injury.

**FIGURE 7:**
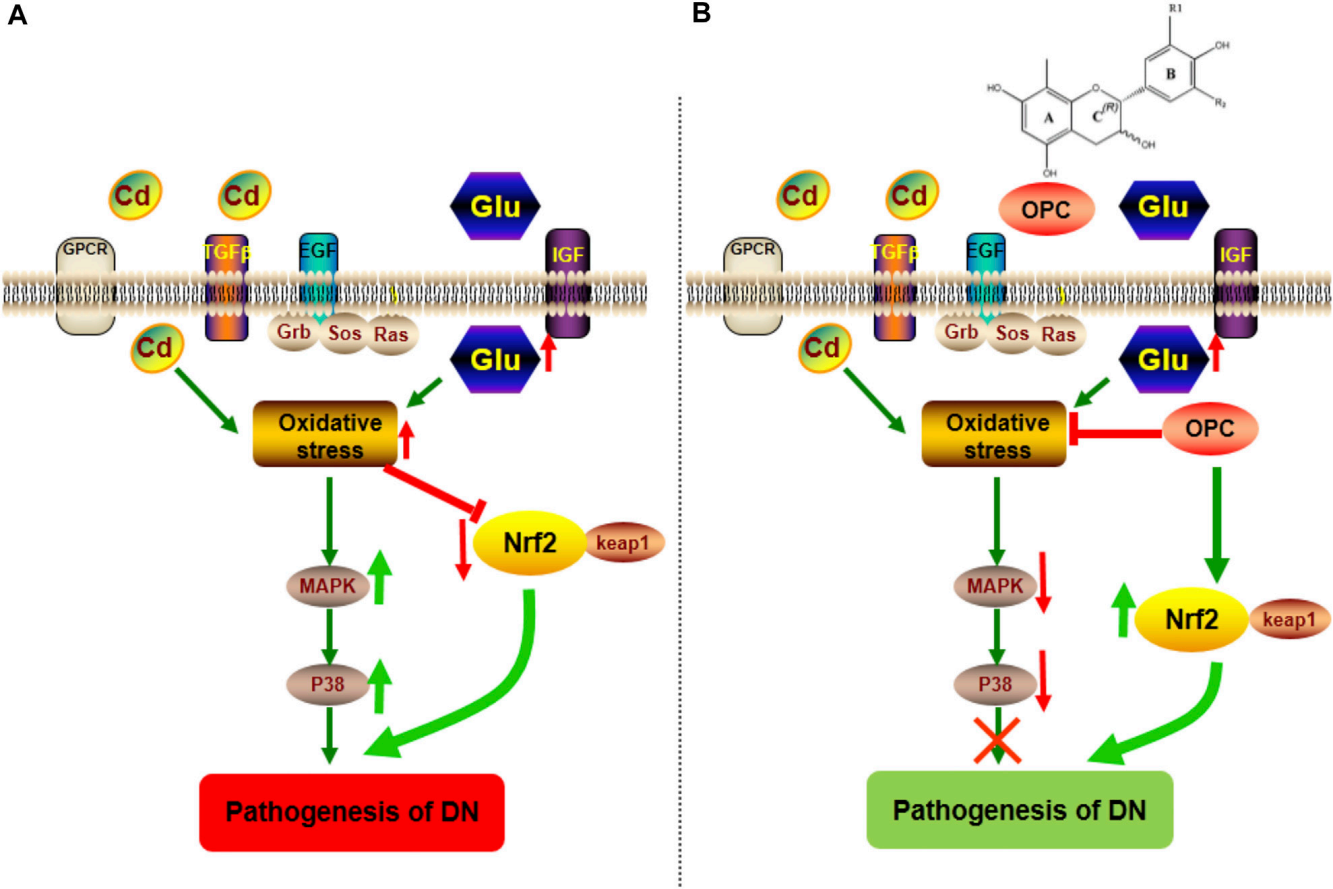
Possible mechanism involved in the pathogenesis of DN and protective effect of OPC. **(A)** The combination of Cd and HFSD can induce the increase of oxidative stress, which activate the expression of p38 MAPK pathway, moreover, block the effect of Nrf2/Keap1 pathway. **(B)** The treatment of OPC can downregulate the expression of p38 MAPK pathway and activate Nrf2/Keap1pathway to exert its protective effect against the progressive of DN.

MAPK functions as a key point in several biochemical processes and participates in cellular processes such as proliferation, differentiation, and apoptosis. p38 MAPK is an important member of MAPK that plays a pathological role in diabetes and result in podocyte apoptosis (Susztak et al., 2006; Chuang et al., 2007). Hyperglycemia is known to activate p38 MAPK (Igarashi et al., 1999; Adhikary et al., 2004), which is associated with the apoptosis of pancreatic β-cells. The p38 pathway is also activated in the renal cortex following podocyte decrease, albuminuria, and glomerulosclerosis during early DN (Wang et al., 2018b). Nrf2 signaling pathway played a critical role in the pathogenesis of renal disease including DN (Chen et al., 2016; Chen et al., 2017; Chen et al., 2019a; Feng et al., 2019a; Wu et al., 2021). A decline in Nrf2 activity leads to the decreased transcription of several antioxidant enzymes and an increased accumulation of ROS. Nrf2 activation not only mediates glucose metabolism and glycogen formation but also plays a role in alleviating oxidative stress by neutralizing ROS and decreasing oxidative damage to the kidneys. Moreover, Nrf2 can negatively regulate TGF-β1 activity (Zheng et al., 2011). Increased evidence has indicated that a number of natural products improve renal injury by activating the impaired Keap1/Nrf2 signaling pathway (Chen et al., 2019b; Feng et al., 2019b; Chen et al., 2019c; Wang et al., 2020). OPC can activate Nrf2 expression associated with the MAPK pathway (Bak et al., 2012). We found that OPC could protect against Cd-induced toxicity by counteracting oxidative injury *via* regulation of the p38 MAPK pathway coupled with Keap1/Nrf2 pathway. The functional group at R2 moiety on OPC appeared important to the inhibitory action of MAPK-related signaling pathway, because prodelphinidin B2 3,3 di-O-gallate, having two galloyl moieties, showed strongest effect while prodelphinidin B2, having no galloyl moiety, failed to show such inhibitory effect (Hou et al., 2007). Our results indicated that targeting of the p38 MAPK and Keap1/Nrf2 signaling pathways might be involved in the pathogenesis of DN. Moreover, OPC triggered a complex crosstalk between p38 MAPK and Keap1/Nrf2 signaling pathway *via* modulating oxidative stress (Figure 7).

## Conclusion

To summarize, we found that OPC from grape seeds exhibit morphological and functional protection in Cd-induced DN and its progression. The protective effects of OPC may be attributed to multidimensional aspects, including the regulation of oxidative-antioxidative status (LPO, PCO, SOD, and GSH), metal-binding ability (Cd in kidney and urine), mediation of the levels of essential elements (Zn, Ca, Cu, and Fe levels in the kidneys), and activation of the p38 MAPK and Keap1/Nrf2 signaling pathways. Based on our findings, it can be reasonably concluded that OPC from grapes can serve as functional foods in preventing the onset of type 2 diabetes mellitus and its complications. However, further studies on the mechanism of action and safe dose of grape OPC are required prior to its use in a clinical setting.

## Data Availability

The raw data supporting the conclusion of this article will be made available by the authors, without undue reservation.

## Abbreviations

ANOVA, one-way analysis of variance; BCA, bicinchoninic acid; Cd, cadmium; DN, diabetic nephropathy; ECL, chemiluminescence; GSH, glutathione; H&E, hematoxylin-eosin; HDL-C, high-density lipoprotein-cholesterol; HFSD, high fat and sugar diet; i.p.,intraperitoneally; LDL-C, low-density lipoprotein-cholesterol; mALB, microalbumin; MDA, malondialdehyde; NO, nitric oxide; OPC, proanthocyanidins; PAS, periodic acid-Schiff; PCO, protein carbonyl; PVDF, polyvinylidene difluoride; SOD, superoxide dismutase; TC, total cholesterol; TG, triglyceride.
