# Correction: Proanthocyanidins protect against cadmium-induced diabetic nephropathy through p38 MAPK and Keap1/Nrf2 signaling pathways

**DOI:** 10.3389/fphar.2026.1842414

**Published:** 2026-04-29

**Authors:** Pin Gong, Peipei Wang, Sihui Pi, Yuxi Guo, Shuya Pei, Wenjuan Yang, Xiangna Chang, Lan Wang, Fuxin Chen

**Affiliations:** 1 College of Food and Biotechnology, Shaanxi University of Science and Technology, Xi’an, China; 2 School of Chemistry and Chemical Engineering, Xi’an University of Science and Technology, Xi’an, China

**Keywords:** cadmium, diabetic nephropathy, proanthocyanidins, Keap1/Nrf2 signaling pathway, p38 MAPK signaling pathway

There was a mistake in Figure 4 as published. The wrong image was uploaded as Figure 4A (Keap1 protein band) during the figure assembly process. The corrected Figure 4 appears below.

**FIGURE 4:**
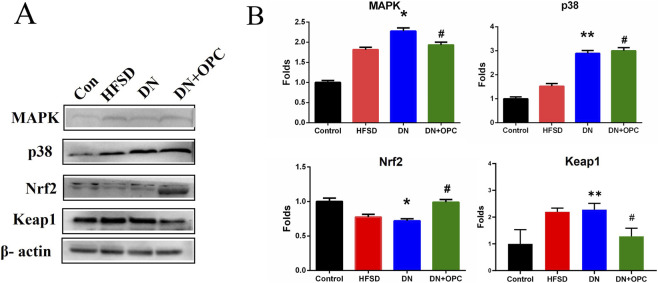
Western blot analysis. **(A)** Protein Bands. **(B)** Quantitative analysis. Data were repeated three times. *p < 0.05, **p < 0.01, compared with control group and ^#^ *p* < 0.05 compared with DN group.

The original article has been updated.

